# Abducens Palsy and Anosmia Associated with COVID-19: A Case Report

**DOI:** 10.22599/bioj.167

**Published:** 2021-01-18

**Authors:** Jessica E. Francis

**Affiliations:** 1 Royal Hallamshire Hospital, GB

**Keywords:** COVID-19, diplopia, anosmia, abducens palsy, neurological

## Abstract

**Aim:**

To report the case of acute isolated abducens nerve palsy and anosmia in a healthy 69-year-old female following SARS-CoV-2 infection.

**Method:**

This is a case report of a previously healthy 69-year-old Caucasian female who presented to the emergency eye centre with a four-day history of binocular, horizontal diplopia eight days after testing positive for SARS-CoV-2 infection. Anosmia was her isolated symptom of COVID-19.

**Results:**

The patient was diagnosed with left abducens nerve palsy. Aetiology was presumed to be post-viral as the patient was not diabetic and had no pre-existing microvascular risk factors. Diplopia resolved within 3.5 weeks. Measurements confirmed complete spontaneous recovery of the abducens palsy within 6 weeks.

**Conclusion:**

Cranial nerve palsies may constitute part of the neurological spectrum of COVID-19 disease. This case report aims to raise awareness amongst clinicians of coronavirus-induced neurological symptoms. Research suggests SARS-CoV-2 infection can trigger an aberrant immune response in some individuals, causing inflammatory nerve damage leading to anosmia and neuropathy. This case report supports the hypothesis that direct or indirect virally mediated injuries along the routes of the cranial nerves can cause neuropathy and olfactory dysfunction. The longer latency effects of COVID-19 infection are not well understood. The long-term rehabilitation of patients exposed to COVID-19 is a major public health concern requiring multidisciplinary expertise. This case report highlights the value of the Orthoptist in the diagnosis and care of patients experiencing neuropathy following COVID-19 exposure.

## INTRODUCTION

In December 2019, an unexplained outbreak of pneumonic cases emerged from Wuhan, Hubei Province China. The disease was confirmed to be caused by the novel coronavirus, severe acute respiratory syndrome coronavirus 2 (SARS-CoV-2) (Li X et al. 2020; Zhu et al. 2020). The infection spread rapidly, and in March 2020 the World Health Organisation (WHO) declared the disease a world-wide pandemic (WHO 2020).

SARS-CoV-2 infection has been reported to produce symptoms ranging from asymptomatic disease or mild cough to fatal acute respiratory distress syndrome (ARDS), severe pneumonia, multiple organ failure and death (Huang et al. 2020). Recent reports have described neurological involvement in COVID-19, affecting the central nervous system (CNS), peripheral nervous system (PNS) and muscle, as well as causing olfactory dysfunction (Asadi-Pooya & Simani 2020; Mao et al. 2020). Incidences of: Guillain-Barre syndrome, Miller-Fisher syndrome, large vessel stroke and isolated cranial neuropathies have recently been reported in the setting of confirmed SARS-CoV-2 infection (Belghmaidi et al. 2020; Ben-David et al. 2020; Dinkin et al. 2020; Falcone et al. 2020; Faucher et al. 2020; Gutiérrez-Ortiz et al. 2020; Oxley et al. 2020; Toscano et al. 2020). To date, there are few case reports describing isolated cranial nerve palsies. This case report aims to expand upon the current literature, suggest possible infection mechanisms for post-viral neuropathies in the context of COVID-19, and highlight the value of the Orthoptist in managing these patients.

## CASE REPORT

In October 2020, a 69-year-old Caucasian female developed acute, binocular, horizontal diplopia eight days after testing positive for SARS-CoV-2 infection. A non-smoker with no known generalised hypertension or diabetes, her only medications were Bimatoprost, (Lumigan) taken one drop into both eyes daily for ocular hypertension, and Sertraline. She reported no history of childhood strabismus. Interestingly, the patient reported a similar episode of diplopia subsequent to flu eight years ago. This resolved spontaneously. No medical records were available of this previous episode as no ophthalmological assessment was carried out at the time.

The patient reported no headache, fever or respiratory symptoms. Anosmia was her sole symptom of COVID-19 infection. The patient was reviewed by the Orthoptist. Ocular motility examination revealed a -1 under action of the left lateral rectus on laevoversion. Alternate prism cover test for distance fixation at 6 m measured a left esotropia of 10Δ in primary position, 7Δ left esotropia on dextroversion and 18Δ left esotropia on laevoversion. The patient reported diplopia in primary position, dextroversion and laevoversion at 6 m. Alternate cover test at 1/3 m revealed a slight esophoria with delayed recovery, this measured 10Δ esophoria. These findings are consistent with left abducens nerve palsy. Her best corrected visual acuity was normal; it measured –0.100 (6/4.8) in the right eye and 0.020 (6/6-1) in the left eye on the ETDRS (Early Treatment Diabetic Retinopathy Study) chart at 4 m (WHO 2019). Pupils were equal and reactive to light. There was no afferent pupillary defect. An eight-dioptre base out Fresnel prism was fitted to the patient’s glasses to relieve diplopia. The patient was also reviewed by an Ophthalmologist in the Emergency Eye Centre. No further investigations were ordered.

The patient was assessed by the Orthoptist 5 weeks later. Left abducens nerve palsy had completely resolved. She reported no diplopia in any gaze position. The patient reported complete resolution of diplopia within 3.5 weeks of onset. Ocular motility was full. Alternate prism cover test for distance fixation at 6 m measured no deviation in primary position, 3Δ esophoria on dextroversion and 2Δ esophoria on laevoversion. Alternate cover test revealed a slight exophoria at 1/3 m with good recovery, this measured 4Δ exophoria. BSV responses for near and distance were recorded using the ‘Worth 4 dot test’. This case has demonstrated the value of the Orthoptist in relieving symptomatic diplopia and advising on prognosis for recovery in post-viral neuropathies.

Two additional cases of transient abducens nerve palsy in two young otherwise healthy patients were noted in May 2020. Both patients reported clinically suspicious symptoms of COVID-19 in the weeks preceding the development of the nerve palsy. Unfortunately, neither individual met government testing criteria at the time of onset so real-time reverse transcription polymerase chain reaction (RT-PCR) tests were not performed, therefore COVID-19 diagnosis was unconfirmed. Similarly, antibody testing was unavailable for the general public at this time. Moreover, anosmia was not listed as an official symptom of COVID-19 by the UK government until 19^th^ May 2020 (Department of Health and Social Care, 2020). Since May 2020, understanding of COVID-19 presentation has developed; consequently, more symptoms are formally recognised. Patient A was a healthy 47-year-old Caucasian female schoolteacher who presented with isolated left abducens palsy. She reported symptoms of sore throat, fatigue, shortness of breath, headache, dizziness and nausea seven weeks prior to the diplopia onset. Diplopia fully resolved in primary position within one month. Residual esophoria was present on laevoversion and dextroversion. Patient B was a healthy 38-year-old male of Chinese ethnicity who presented with isolated right abducens palsy. He worked as a caregiver in a residential home setting with recent confirmed COVID-19 outbreak. He reported symptoms of dry cough, sore throat and malaise two to four weeks prior to onset of diplopia. Diplopia in primary position resolved within one month. Ocular motility was full. In both cases, ophthalmological assessment was unremarkable and MRI scans normal. In the absence of a space occupying lesion and demyelination, post-viral aetiology was suggested. Both patients worked in high-risk occupations, hence COVID-19 exposure is highly possible. Notwithstanding, this evidence is speculative in the absence of a confirmed positive COVID-19 test.

## DISCUSSION

The abducens nerve (sixth cranial nerve) is a motor nerve supplying the lateral rectus muscle. Clinical presentation manifests as horizontal diplopia, limitation of abduction and an eso-deviation in primary position, measuring greater for distance than close proximity. The route of the abducens nerve is long and consequently more susceptible to insult. Abducens nerve palsy is the most common isolated cranial nerve palsy (Elder et al, 2016). The aetiologies of abducens palsy are diverse, including microvascular ischemia, space-occupying lesion, trauma, viral and bacterial infections, stroke, inflammation and idiopathic origin (Bhardwaj et al. 2013; Choi et al. 2019; Sharma & Biswas. 2010). The association between viral infection and cranial neuropathy is well reported in the literature. In particular, abducens nerve involvement has been linked with confirmed viral infections caused by: Cytomegalovirus, Epstein-Barr virus, Herpes Zoster, Lyme Disease, Measles, Dengue Fever and Scrub Typhus (Bhardwaj et al. 2013; Greco et al. 2006; Shin et al. 2007; Shivanthan et al. 2012; Werner et al. 1983).

## MECHANISM

Previous research into SARS-CoV infection pathways and mechanisms suggest neurological symptoms occur due to viral invasion via the olfactory nerve (first cranial nerve) proliferating to the thalamus and brainstem (Li Y et al. 2020). This may explain the high prevalence (47%) of anosmia in patients with COVID-19 (Borsetto et al. 2020). The understanding of the precise mechanism of coronavirus-induced neurological symptoms is currently evolving. Aberrant immune response may induce neurological injury from pro-inflammatory cytokines (Gutiérrez-Ortiz et al. 2020; Wang et al. 2009). This case report presents anosmia and an isolated abducens nerve palsy occurring within the setting of SARS-CoV-2 infection. This suggests that direct or indirect virally mediated injuries along the routes of the olfactory and abducens cranial nerves, respectively, could explain the presentation of these concurrent symptoms.

Spontaneous recovery of abducens palsy occurred within six weeks in all three cases. The transient nature of the cranial nerve palsies described in this report is in keeping with other case reports. Faucher et al. (2020) reported the spontaneous resolution of left oculomotor cranial nerve within seven days in a 21-year-old male with COVID-19 infection. Gutiérrez-Ortiz et al. (2020) reported a 39-year-old male with bilateral abducens palsy and COVID-19 infection. He was treated with acetaminophen and the nerve palsy resolved within two weeks. Similarly, Belghmaidi et al. (2020) reported a 24-year-old female with left oculomotor cranial nerve palsy. She was treated with chloroquine and azithromycin, the nerve palsy completely resolved within six days.

## LIMITATIONS

The occurrence of isolated abducens palsy following SARS-CoV-2 infection in this 69-year-old female patient could be simply coincidental and unrelated to the reported anosmia. Indeed, microvascular ischemic aetiology could be involved in this patient despite the absence of any known preceding microvascular risk factors. The most common cause (83%) of abducens nerve palsy in patients aged over 50 years is microvascular ischemia (Choi et al. 2019). The typical recovery time for isolated microvascular nerve palsy is eight to twelve weeks (Capó et al. 1992). In contrast, complete resolution of the abducens nerve palsy in this case occurred within 6 weeks; this supports post-viral aetiology. Interestingly, microvascular events are a known secondary complication of severe COVID-19 disease; however, this patient was largely asymptomatic (Roberts et al, 2020).

Further case studies are necessary to confidently support causality. The lack of testing availability in the UK during the early stages of the pandemic produces difficulties when retrospectively reviewing potential COVID-19 cases and the longer-term implications of infection. In the early stages of the pandemic, healthy individuals who experienced mild COVID-19 symptoms and did not require hospital admission did not qualify for testing. Nevertheless, these patients may still present with ophthalmological and neurological symptoms after resolution of characteristic constitutional and/or respiratory COVID-19 symptoms. At present the true extent of infection is unclear.

## CONCLUSION

The long latency effects, neurological manifestations, infection pathways and mechanisms of COVID-19 are currently not well understood. Coronavirus induced anosmia could be explained by invasion and subsequent immune-mediated injury to the olfactory nerve (cranial nerve I) (Gutiérrez-Ortiz et al. 2020; Li Y et al. 2020; Wang et al. 2009). This infection mechanism could affect other cranial nerves and produce isolated nerve palsies. Infection is a known aetiology of isolated abducens nerve palsy, thus SARS-CoV-2 infection is a plausible cause for the occurrence of isolated abducens nerve palsy with associated anosmia described in this case report. The rapid recovery of abducens palsies reported here also support the theory of viral aetiology.

Further case reports are necessary to reveal the full spectrum of clinical manifestations of COVID-19 infection. This case report will raise awareness of the neurological signs and potential longer latency effects of COVID-19. As confirmed cases of COVID-19 rise worldwide, Ophthalmology outpatient departments may see an increase in cranial neuropathies in otherwise healthy individuals, following COVID-19 infection. The long-term effects of the global pandemic are largely unknown and a major public health concern. This report highlights the value of the Orthoptist in the diagnostic and therapeutic rehabilitation of these patients.
